# A heuristic model for collaborative practice – Part 1: a meta-synthesis of empirical findings on collaborative strategies in community mental health and substance abuse practice

**DOI:** 10.1186/s13033-020-00376-5

**Published:** 2020-06-09

**Authors:** Rolf Sundet, Hesook Suzie Kim, Bengt Eirik Karlsson, Marit Borg, Knut Tore Sælør, Ottar Ness

**Affiliations:** Universitetet i Sorost-Norge, Drammen, Norway

**Keywords:** Meta-synthesis, Collaboration, Mental health and substance abuse, Community mental health and substance abuse practice, Service user/professional collaboration, Collaborative strategies, Turn-taking

## Abstract

**Background:**

Collaboration has become a cornerstone for healthcare practice in recent decades resulting in the efforts at international and national levels to integrate the concept into healthcare practice and services. However, there is a paucity of research delineating strategies for professionals to apply in collaborative practice with clients in general as well as in mental health and substance abuse (MHSA) care.

**Methods:**

The method applied in this paper is a form of qualitative meta-synthesis referring to the integration of findings from multiple qualitative studies within a program of research by the same investigators. Eighteen empirical papers with the focus on community MHSA practice and recovery-orientation with relevance to the service user–professional relationship in MHSA practice were included in this meta-synthesis.

**Results:**

Three types of processes of collaboration specified by meta-themes were identified. The meta-themes of the interactive-dialogical process type include (a) maintaining human relationship, (b) walking alongside, (c) information sharing, (d) seizing the present moment, (e) taking the perspective of the other, and (f) aligning/scaffolding. The meta-themes of the negotiated-participatory engagement type include (a) feedback-informing process, (b) putting differences to work, (c) negotiated partnering, (d) accommodating user participation, and (e) addressing the tension between help and control. The meta-themes of the negotiated supportive process type are (a) helping in context, (b) coordinating, (c) pulling together, (d) advocating, and (e) availing. These meta-themes are strategies for collaboration applicable in MHSA practice.

**Conclusions:**

This meta-synthesis of collaborative processes found in community mental health practice points to the possibility of developing a set of repertoires of practice for service user/professional collaboration, especially in community MHSA practice.

## Introduction

Collaboration has become a cornerstone for healthcare practice in recent decades resulting in efforts at international and national levels to integrate the concept into healthcare practice and services [1–3]. The report by the UN’s Human Rights Council [4] specifies the “participation” of everyone in decision-making at the legal, policy, community and health service level as one of the criteria for the “right to mental health framework.” Furthermore, the mandate emphasizes the critical feature of “the freedom to control one’s own health and body” linked to “the right to liberty, freedom from non-consensual interference and respect for legal capacity” in relation to mental health and mental health service [4, p. 8]. Collaboration and collaborative practice advocated in these mandates refer to their significance in the entire spectrum of mental health and substance abuse (MHSA) care at the societal, community, healthcare, and individual levels not only in terms of the provision of healthcare but also in relation to policy development, service designs and distribution, and accessibility. One of the major issues regarding collaboration in MHSA care is client–professional collaboration, especially in light of the historical context of power asymmetries that existed and are still pervasive in clinical services. The scholarly attention to client-professional collaboration has not been rich although it is the client-professional collaboration that is most critically and directly affecting user outcomes especially in MHSA care in which relationships between a user (or family) and a professional are the major modes of service. The terms such as therapeutic alliance, helping relationships, professional–patient partnership, relational recovery, and involving patients in care have been considered important in MHSA practice as well as in general healthcare practice, however they do not embrace the comprehensive features of collaboration that encompasses “sharing common grounds,” “negotiation,” and “working together.” Clinical encounters between service users and professionals in MHSA care are the occasions at which relationships are established and often maintained over time affecting therapeutic processes and user outcomes. Collaboration in this context refers to (a) arriving at mutually agreed values, goals, and positions, and (b) working in partnership with each other arriving at goals. The processes of collaboration in client–professional relationships have been identified in general terms as dialogic and sharing [5, 6], shared problem-solving and decision making [7, 8], recovery-orientation [8–10] and partnership and participatory engagement [11–13]. However, there is a paucity of research delineating strategies for professionals to apply in collaborative practice with users in general as well as in MHSA care.

The critical importance in delineating such strategies is the perspectives of participants in relationships—in this case, the perspectives of users, family members, and professionals. Our team of researchers at the Centre for Mental Health and Substance Abuse at University of Southeastern Norway (the CMHSA-USN) with an interest in community MHSA practice has published a rich set of qualitative studies investigating the perspectives of participants regarding interactive phenomena in client–professional relationships and MHSA practice during the past 15 years. This report is a meta-synthesis of these reports to delineate strategies central to the collaborative process in MHSA care. Although such strategies as *listening*, *sharing information*, and *constraint*-*free communication* have been considered collaborative, there is a paucity of studies that identify interactive strategies of collaboration in the literature. Furthermore, no systematic synthesis of collaborative strategies integrating the perspectives of participants is found in the literature. This meta-synthesis, therefore, presents a comprehensive set of collaborative strategies that are applicable in MHSA care addressing the research aim to enrich the knowledge base for collaborative practice in community MHSA care.

A precursory clarification regarding the use of specific terms in this paper is in order. Among the terms such as client, patient, service user, and consumer we use the term “person” or “user” to refer to the citizen in need of or using healthcare service for MHSA care, while among the terms such as clinician, professional, therapist, or service provider, we use the term “professional” to refer to the person providing clinical, professional health care services directly to users. In addition, we use the term “clinical practice” to encompass the work of healthcare that involves therapy, care, and services for people in need of health care.

## Method

The method applied in this paper is a form of qualitative meta-synthesis. The term, qualitative meta-synthesis, has various meanings, refers to variant approaches, and is often applied in systematic review studies. The qualitative meta-synthesis applied in this paper is in line with the first kind of syntheses identified by Sandelowski, Docherty, and Emden [14] referring to the integration of findings from multiple qualitative studies within a program of research by same investigators. The purpose of this approach in this paper is to arrive at a theoretically meaningful synthesis about strategies having the common theme of “collaboration” through the integration and comparison of the qualitative empirical material we have accumulated in our studies of community mental health practice. The procedural steps adopted reflect the seven steps identified by Noblit and Hare [15] for meta-ethnography, which include (a) getting started, (b) deciding what is relevant to the initial interest, (c) reading the studies, (d) determining how the studies are related, (e) translating the studies into one another, (f) synthesizing translations, and (g) expressing the synthesis.

The studies included in this meta-synthesis are carried out by a team of researchers in a program of research within an institute of mental health care practice and research (at the CMHSA-USN). The focus of our synthesis was the processes of collaboration in mental health practice. Therefore, the first four steps of this method have been well established within the group. The application of this qualitative meta-synthesis thus encompasses the three last steps beginning with the fifth step of translating the studies into one another, synthesizing those translations, and expressing the synthesis. While meta-ethnography and meta-synthesis, in general, are oriented to “synthesizing” researchers’ interpretations of qualitative data in original studies, which are social constructions “built into accounts of methods, in the theories used, in the researchers’ worldviews” [16, p. 3], this meta-synthesis did not have to deal with the issue of consolidating different perspectives or worldviews. This meta-synthesis began with the foreknowledge of our perspectives, methods applied, and our world-views, which align with the epistemological stance of phenomenological-interpretive and critical perspective.

For the fifth step of translating the studies into one another, the themes and concepts from each study with their descriptors were identified, compared and contrasted reflecting also upon the relevant literature. In addition, the authors identified additional collaborative strategies that were alluded but not identified as specific themes in the publications by returning to the papers and original empirical material. With results from the fifth step, the sixth step involved consolidating, integrating, and augmenting the themes and concepts from the studies into meta-themes in explicating collaborative strategies applying discernment and creativity of the researchers critical in qualitative synthesis [16]. This paper provides the results of this meta-synthesis describing the meta-themes as the key strategies of collaboration to be the base for elaborating a collaborative practice model for MHSA practice.

## Results

Eighteen empirical papers by this research team at the CMHSA-USN published from 2004 to 2017 with the focus on community MHSA practice and recovery-orientation with relevance to the service user–professional relationship in MHSA practice were included in this meta-synthesis. Table 1 lists these studies in chronological order of publication in terms of the methods used, research participants, and themes/concepts applicable to explicating collaborative strategies. All of these studies applied qualitative methods, mostly focus-group method and in-depth interviews with the research participants that included service users, family members or significant others of service users, and professionals. The analytical methods applied in these studies were descriptive and/or interpretive.

**Table 1:** The list of published articles by the team on mental health and substance abuse practices related to collaboration and recovery-orientation used for the meta-synthesis

Publication	Research questions	Method	Research participants	Themes and Meanings
Borg and Kristiansen [17]	The aim of this research is to understand the characteristics of helping relationships in mental health services, including the ways in which recovery-oriented professionals can most effectively collaborate with service users.	Individual interviews	Persons with lived experience of severe mental illness	Discover fellow humanity (Being seen as a person; Being seen as “both-and” and” ill-and-well” Available everyday helpers (Just being there—finding the time to be there) Experiencing what is “therapeutic” (Inspiring hope and courage; Being trustful) Breaking the rules (Being the personal relationships as the base for professional ones; Being supportive with necessary control)
Borg et al. [18]	This article focuses on the role that material resources, particularly having a house or an apartment, as well as the associated meanings that make such places ‘‘home,’’ play in processes of recovery from severe mental illness	Individual interviews	Persons with lived experience of severe mental illness	Dealing and living with major struggles: Poverty, unemployment, substandard living and homelessness Importance of having a home: As a place of growth, development and control A need for balancing the need for socialization with privacy A home as a secure base
Karlsson et al. [19]	How do CRHT team members understand and negotiate their understanding of a person experience a mental health crisis? The aim of this paper is to gain a deeper understanding by exploring how the team members reflect upon the experiences of their understandings and negotiations related to mental health crisis	Focus group interviews	Professionals	Description of mental crisis and which implications this understanding has for working in teams Understandings of crisis Negotiations on crisis
Borg and Davidson [20]	The present study was intended to contribute to a growing body of knowledge that attempts to explore, understand, and address severe mental health problems within the context of the person’s everyday life	Individual interviews	Persons in recovery from severe mental illness	Themes for recovery: Begin normal Just doing it Making life easier Being good to oneself Implications for user/professional relationships: Remaining open to opportunities which present themselves Supporting users’ efforts to function as citizens
Borg et al. [21]	To elicit and learn from service users’ experiences about the subjective meanings of crisis and what kind of help will be most effective in resolving mental health crises	Focus group interviews	Persons with experiences of mental health crisis	Experiences of mental health crisis: Crisis as multifaceted and varied experiences Losing the skills and structure of everyday life The complexities involved in family support Implications for practice: Supporting person-in-context perspectives and person-centred ways of working
Sundet [22]	This qualitative study examined how a group of families and their therapists described helpful therapy	Individual and family interviews	Therapists, and users of family therapy	Helpful therapy is: The helpful relationship, the helpful participation and the helpful conversations Therapists: “To get a taste of it” (Sharing experiences; Participating, attaining mutual definitions; blurring the differences) “The lingering conversation and the big toolbox (Questioning; Lingering; Content; Nuancing the nuances) “To be where people are” (Listing, talking seriously, and believing; Being flexible; Generosity) Family members (Users): The helpful conversation (Asking questions, giving time, and structuring the work; Giving and receiving feedback; Reformulation) The helpful participation (Using professional knowledge; Having many possibilities; Understanding though participation) The helpful relationship (Generating collaboration; Giving of oneself; Fighting violation, disparagement, and degradation)
Ervik et al. [23]	The aim of the study was to explore and interpret milieu therapists’ experiences of collaboration between employees and residents at a low threshold accommodation center for homeless men	Focus group interviews	Professionals	Unpredictable and challenging weekdays, and experiences with collaboration was gathered in the following: The knowledge and experiences of the professionals The spontaneous and informal Conditions employees do not control Grasping the moment expressed understanding of wholeness about collaboration between professionals and patients
Borg et al. [24]	The objective of this study was to explore and learn from relatives’ experiences about the subjective meanings of crisis and meaningful and efficient crisis support	Focus group interviews	Family members	Relatives experience of mental crisis: Experiences of rejection and responsibility Living with contradictions The art of balancing openness
Sundet [25]	The aim of this qualitative study was to explicate how therapists describe and evaluate the use of two measures, the Session Rating Scale and the Outcome Rating Scale, used as feedback tools.	Individual interviews	Professionals	Therapist perspectives on the use of feedback from patients/families with feedback scales as conversational tools Conversational types: getting feedback, create structure, make discoveries, separation between person and problem, getting results Upholding collaboration
Karlsson et al. [26]	The aim was to explore service users’ and professionals’ subjective experiences of attending the study course together, and the following two research questions were asked of patients and therapists: (1) How would you describe your experiences in relation to the content and the processes of the study course? (2) In what ways has the study course had an impact on your own awareness and role in the process of empowerment and recovery?	Focus group interviews	Patient-therapist pairs	Recognizing common humanity and common strength (Sharing the humanity being equals; Being together in the sense of community) Being accepted as a person (Respect for individuality) An inviting control-free zone (Letting go of controls; Working together and partaking in activities together) Doing things differently (begin free of contextual constraints)
Borg and Karlsson [27]	The objective of the present paper is to explore philosophical ideas and experiences of a home and the opportunities and dilemmas of home treatment	Two studies: 1. Individual interviews 2. Focus group interviews	1. Service users 2. Professionals	The home as an arena for treatment, rehabilitation and caring Self-control, safety, beneficence and autonomy in both professional and patient
Ness et al. [28]	The purpose of this paper is to describe parents’ experiences of collaboration with mental health practitioners when working with young adults with co-occurring mental health and substance use problems	Focus group interviews	Clinicians in CMH care	Walking along side (Be with them) Maintaining human relationships (Be there; Continuing with relationships and not rejecting) Maneuvering relationships and services (being coordinators; Being advocates)
Sundet [29]	The aim of this study is to explain how families within mental health for children and adolescents evaluate and describe the use of two measures, the Session Rating Scale and the Outcome Rating Scale, as feedback tools	Interviews (CQR)	Family members receiving MHC	Practice using ORS and SRS Confirmation and confirmation of functions Difficulties (Difficulties associated with the lack of information with administration of the scales; Difficulties associated with the form of the scales; Difficulties associated with special situation and consequences of having the ratings)
Ness et al. [30]	The purpose of this paper is to describe parents’ experiences of collaboration with mental health practitioners around young adults struggling with co-occurring mental health and substance use problems	Focus group interviews	Family members/significant others of young adult users	Negotiating partnerships (Being appropriately involved through negotiated involvement) Incomprehensive services (Helping to understand the dynamics of services) Being the users’ advocates
Sundet et al. [31]	Drawing from experiences of a family-based practice the article seeks to give in-depth specification of processes embedded within collaborative practice that is built around turntaking	Clinical encounters	Family therapy session	Turn-taking Negotiating for common goals (Being aware of one’s own goals and expanding understandings of one another; Moving with the differences) Putting differences to work (Moving along)
Soggiu and Biong [32]	The purpose of the study was to investigate and describe the experiences of social workers with overdoses and overdose deaths. The research question was: How do social workers describe their experiences with overdoses and overdose deaths of clients with an opiate dominated misuse of injections.	Focus group interviews Individual interviews	Professionals	Experiences of working with overdoses and death from overdoses Difficulties in planning the work What the clients needed Life is not lived within the healthcare system
Ness et al. [33]	The aim of this article is to explore and describe the experiences of young adults with co-occurring mental health and substance use problems perspectives on collaborative practices with practitioners.	Individual interviews	Young adult service users	Don’t fix me or judge me Someone to sort issues out with Not giving up Practical help
Sundet [34]	The chapter summarizes experiences with the use of two measures, the Session Rating Scale and the Outcome Rating Scale, as feedback tools, with the aim of explicating how a family team in mental health for children and adolescents has come to understand their work with feedback from the families	Summery of empirical findings and clinical experiences	Family members receiving MHC Professionals	ORS and SRS as conversational tools To be both client—directed and outcome-informed Service users at the core of therapeutic work To follow the client and be challenged by data

All of the studies included in this meta-synthesis were carried out in the context of community mental health practice, and the mental health problems experienced by the user-participants, in general, represent those found commonly in this context such as acute mental health crises not requiring inpatient care, long-term mental health and substance abuse problems requiring continuing care, and other mental health issues related to psychological and social functioning.

The synthesis of the themes and concepts found in these works involved consolidating similar themes and specifying them into meta-themes by comparing the themes and their meanings. Some of the themes extracted in singular publications were also specified as meta-themes when they were interpreted to be critical collaborative strategies. This analysis led to a three-level explication of the results: (a) the first level involving the synthesizing process to extract the meta-themes as a comprehensive set of collaborative strategies drawn from the empirical studies, (b) the second level identifying the over-arching process of turn-taking as the central concept for collaborative work, which is both integrative and undergirding the meta-themes emerged from the synthesizing work, and (c) the third level which is analytically oriented categorizing the explicated meta-themes into three distinct process types in terms of interactive-dialogic processes, negotiated-participatory engagement processes, and negotiated-supportive processes. Table 2 shows the meta-themes and their meanings in the three process types.

**Table 2:** Meta-themes for collaborative practice

Processes of collaboration	Meta-themes	Major meanings
Interactive-dialogic processes	Maintaining human relationship	Establishing social connectedness
	Walking alongside	Being a companion with equal footing
	Information sharing	Working with what is present
	Seizing the present moment	Offering information with the other openly
	Taking the perspective of the other	Opening up for and accepting differences
	Aligning and scaffolding	Fitting together the strengths and weaknesses of oneself and the other
Negotiated-participatory engagement processes	Feedback-informing process	Using feedback for information sharing and negotiation
	Putting differences to work	Accepting the differences and putting those differences to work constructively
	Negotiated partnering	Working out what to share and how to share the work
	Accommodating user participation	Promoting and enhancing user participation
	Addressing the tension between help and control	Mediating the tension
Negotiated-supportive processes	Helping in context	Helping that is specific to situations
	Coordinating	Coordinating services and resources
	Pulling together	Forging together for social participation
	Advocating	Campaigning for users
	Availing	Making clear regarding what, how, and when of available help

*Turn*-*taking* as an over-arching process emerged from this analysis as the principal conceptual base upon which all of the strategies and processes extracted as the meta-themes in the analysis built their special features. Turn-taking is the pattern of back-and-forth acts and processes that happens between two or more interactants characterized by alternating responses [35, 36]. This pattern of alternating responses is the starting point and building ground for any joint inter-human and interspecies phenomena. Being together is all about turn-taking. During our life span, this format of alternating responses is realized in different media; from the non-verbal, bodily expressed mutual responses between the infant and caregiver, to any interactional, transactional, communicative, conversational or dialogical event and situation. *Active sharing involvement* and togetherness do not arise without the pattern of turn-taking. In our context with mental health practice, the establishment of turn-taking is decisive. In the following presentation of the meta-themes as the critical categories for the collaborative practice, turn-taking is the implicit principle and is the medium for the repair of breaches and ruptures in interactions.

## Interactive-dialogic processes

Interactive-dialogic processes encompass those strategies and modes of connecting among participants through discursive/dialogic modes as well as corporeally and socially oriented modes of interaction as persons with social roles in specific contexts—which in this work, the context is the clinical encounter. Clinical encounters between the user and the professional involve spending time together talking and interacting for the primary purpose of supporting the person in the path of recovery, taking place not only at clinical service settings but also at other non-service settings such as homes, work settings, or casual environments. Interactive-dialogic processes in clinical encounters involve building relationships and getting to know one another in order to arrive at mutual understandings especially in the context of collaborative practice. From our work, six meta-themes as the collaborative strategies of interactive-dialogic processes are extracted which are: (a) maintaining human relationship, (b) walking alongside, (c) information sharing, (d) seizing the present moment, (e) taking the perspective of the other, and (f) aligning and scaffolding.

### Maintaining human relationship

Maintaining human relationship is the theme that is critical especially in MHSA practice because the user/professional relationships tend to be long-term and continuing. This theme consists of three tenets: (a) clinical encounters are relationship-building which often continue over time [28], (b) service user–professional relationships are based on the shared, common essences as humans as opposed to being “us” different from “them” [26], and (c) human relationships are maintained and thrive when participants recognize and accept the humanity having the same essences and uphold each other’s personal resources and experiences as valuable, especially when professionals acknowledge users or families to have valuable contributions to make with their experiences and resources [30]. Recognizing common humanity and common strength by participants are the essential features of collaborative relationships and equality in the processes of MHSA therapy [26]. Maintaining human relationships in the user/professional relationships implied the commitments for continuing support for service users’ involvement in the clinical process and valuing of service users’ uniqueness, strengths, and possibilities by professionals. It is accomplished through an openness for discussions and unconstrained negotiations. To have a positive and helpful relationship in MHSA care depends on that both users and professionals mutually see and experience each other as persons as the primary condition.

### Walking alongside

Walking alongside refers to interactions that put participants in a same course of progression through establishing a partnership of negotiated dialogues toward a mutually agreed upon destination and direction [28]. The professionals must respect the integrity and uniqueness of the service user by following and laying aside the professional’s beliefs and preferences. The dialogues involve taking the situations, hopes, and dreams of the person as the starting points. Walking alongside implies establishing a good relationship by not taking over the life of the person, and being flexible in responding to her/his needs [17, 28]. Walking alongside is also expressed as the theme of “Don’t fix me or judge me” by young adult users [33]. Walking alongside as a form of collaboration is also evident in working with families with children or adolescents in their MHSA care [22]. Walking alongside also means the availability of quick help in crisis including giving help outside the office hours or at places other than the standard therapeutic settings [30]. Overall, walking alongside is about elevating the position of the persons, families, and networks to be on par with the professionals, getting away from the power differentials between users and professionals. The users and sometimes their family members will have possible blueprints for the courses of living pertaining to mental health problems, and the professional is a knowing companion who can point out guideposts to the users and family members as they walk alongside on the path to recovery. This means that professionals need to be flexible in how to accommodate individual differences.

### Information sharing

Information sharing with users has become the major requirement in the consumer-oriented movement in healthcare and in promoting person-centered care. In this sense, information sharing is the first step in collaborative practice as well since the collaborative practice has to involve informed participants. However, in our studies, users and their families voiced their concerns for not getting the information needed from professionals and other service providers in their clinical/service encounters. Information about the ways services are provided or regarding the complexity in the healthcare system as well as regarding how to navigate in getting needed help seemed not to have been given sufficiently to service users or their families [24, 30]. In addition, family caregivers sometimes experienced being rejected or ignored of their needs for information, forcing them to take over additional responsibilities. Professionals also experienced a lack of access to the information within and between services as well [32].

### Seizing the present moment

Seizing the present moment refers to seeing the importance of what is happening on the spot and taking that importance to move forward in interaction, even if that means going away from the planned course of progression. In general, clinical discourses between users and professionals begin and progress in a somewhat routinized, generalized fashion. However, each encounter and its discourse take on a unique stream as the process of turn-taking takes place, and there are moments open for grabbing to get attention. Critical elements of seizing the moments are spontaneity, unexpectedness, and informal and off-the-course happenings [23]. Although possibly challenging, the spontaneity gave opportunities and possibilities for building collaboration and relationships. Part of this was adjusting oneself to the situation and needs of the users. Spontaneity in grasping what is present at a given moment such as the pleasure of managing small tasks of everyday life as a cue to move toward recovery and change seemed valuable even if such a cue may lead to a detour [20]. This means that recovery should not be viewed as a planned, rational, and stepwise process, that is, it is the capturing the essences of the present moment in everyday life situations and to take them as the pivot to move forward with the collaborative work.

### Taking the perspective of the other

“Taking the perspective of the other” is oriented to the mutual understanding that involves seeing the self as the other sees and seeing the world or the matters of the world as the other sees. Although the term, ‘the other’, can be the generalized other or a specific other, in relationships between the user and the professional it is the specific other of the relationship of whose perspective is taken. Ness et al. [30] state that good collaboration with parents is built with the parents seeing the professionals as a resource and having openness for discussions and negotiations with them. Sundet [22] found that both therapists and families underline what one of the therapists referred to as “getting a taste of it.” In this sense, “taking the perspective of the other” is not only rationally seeing and understanding but actually responding where one`s emotions can be seen and experienced as something similar to what the other is experiencing. Concepts like “resonance” and emotional transport are part of this theme that could enhance collaboration.

### Aligning and scaffolding

The theme of “aligning and scaffolding” refers to the movements of participants in relationships toward each other in order to be on the same footing as a process of adjustment. Aligning is the movement of professionals to be in line with users, while scaffolding is the movement for users to gain better and deeper insights into issues with the support of professionals’ knowledge and experiences, which usually brings users closer towards the professionals’ standpoints. Both are processes aimed at mutual understanding and for establishing a unified stand for supporting persons in their journey in recovery.

In the process of walking alongside, aligning responses and questions from professionals to users lead the users to their own clarification of meanings [28]. Professionals’ alignment and adjustment to users or their families to their needs by slowing down, repeating, or asking questions in different ways seemed critical in applying the routine Outcome Monitoring, especially when they were stuck without progressing further or when there are ruptures in the therapeutic relationships [25, 29]. Implicit in such situations of no-progress or rupture in therapeutic relationships is that they stem from differences between users’ goals, needs, preferences and perspectives and those of professionals, which have not been reconciled, rather than from having wrong ideas or faulty techniques. Although tailoring practices to persons imply that their preferences, perspectives, needs, and ideas are the base for the tailoring, the situations are not necessarily, such that these are given explicit and verbal formulations by users. This means that part of the professional’s task is to help the user to make these explicit. Scaffolding having its orientation in the sociocultural theory of learning involves the dialogical, interactive process of support and guidance through which the person discovers new knowledge and understanding. The person through questions and responses with the professional moves incrementally and progressively from what he or she knows and is familiar with to discover new possibilities, ideas, perspectives, and preferences. Through scaffolding offered by the professional, the user is able to move gradually into new understandings and insights, new knowledge, and new formulations or to be able to clarify what the user means. Service user/professional dialogues of asking questions, getting answers and feedback, and having opportunities for reformulating ideas and understandings were helpful and useful to service users in gaining new understandings or clarifying meanings [22, 28].

## Negotiated-participatory engagement processes

Negotiated-participatory engagement encompasses the processes for collaboration that involve ‘doing things together’ in order to accomplish the work of recovery and of remaining as healthy as possible for the person. It refers to active sharing and negotiated involvement of participants in the work of shared decision-making, goal setting, planning, and actions for recovery. The foundation for negotiated-participatory engagement is the mutual understanding that results from various interactive-dialogic processes in clinical encounters between users and professionals. The core facet of negotiated-participatory engagement processes is “shared decision-making” that involves negotiating, coming to an agreement regarding responsibilities, and finding the basis for complementarity in contributing to the work of recovery and getting/staying well. Shared decision-making addresses what the nature of problems is, what types and courses of collaborative plans should be followed, who should be involved in such collaborative plans and in what ways different people would contribute to this work, and what sorts of resources should be tapped for application in specific situations. In our work, we extracted five meta-themes for this category of negotiated-participatory engagement, which are: (a) feedback-informing process, (b) putting differences to work, (c) negotiated partnering, (d) accommodating user participation, and (e) addressing the tension between help and control.

### Feedback-informing process

The feedback-informing process involves engaging the service user and the professional to join discussions of clients’ views of their own outcomes as the pivot for moving forward with treatment plans and intervention. It has evolved from the process developed in the Outcome Monitoring Feedback Systems (OMFS) that were developed to provide professionals the knowledge of outcomes as perceived by users in order to influence the ways professionals carry out therapies in mental health care especially in following users on a continuing basis. The formal process of joint feedback informed process, therefore, represents the “*active sharing involvement”* as a way to attain collaboration in therapeutic interactions.

Sundet [22, 25, 29] found that the feedback-informing process resulted in better collaboration between users/families and professionals and better outcomes. The user-feedback tools did not simply give information but were used more generally as conversational tools. The tools did not give answers but provided the base for questions and conversations regarding collaborating as well as about other themes that were important to the users/families. These conversations help to verbalize the unsaid and to make shared decisions on what works and how to move on [25, 29]. These tools give opportunities for users to bring matters of their lives as they prefer and want to the forefront of discussions, giving the professionals to understand and respect the persons’ preferences. As conversational tools that elicit questions, they also represent strong imperatives to respect the users’ answers and to follow users’ preferences and choices.

### Putting differences to work

Putting differences to work refers to using differences that exist between the service user and professional in terms of perspectives, goals, and approaches as advantages in moving forward with clinical plans. This implies valuing the differences, that is, differences in understanding, perspectives, ideas, practices, goals, etc., which leads to a negotiated division of labor [31]. This is accomplished through two forms of conversation identified as dialectical and dialogical by Sennet [37]. The dialectic refers to conversations that through differences (thesis and antithesis) lead to something new that all parties can agree upon (synthesis). In the dialogic form there is no such closure, but an increased realization and acceptance that the participants have different ideas, perspectives and actions, and that in the collaborative work these tensions generated by the differences are retained, allowing people to work together within these differences and tensional relationships. Karlsson et al. [26] in the study of participating in a course for empowerment and recovery by user–professional pairs found that getting to know and appreciate the differences in meanings and perspectives of their partners were viewed important in building their relationships and working together within their relationships.

### Negotiated partnering

Negotiated partnering refers to doing things together with clear understandings about different contributions to be made by persons involved in the work through negotiations. The concept of negotiated partnering involved the professionals’ acknowledgment of the value of resources held by persons and their families and putting such resources into use through negotiation. This was most evident in situations involving adult-children and youth as users in which parents with in-depth knowledge about their children had the desire for involvement in the care and at the same time felt constrained by the understanding to let them live their own lives or by professionals’ objections for their involvement [30]. In such cases, the negotiated partnering among the user, the parents, and the professionals was a key as their involvement in the therapeutic process required complementarity and harmony. Professionals are the key players in establishing negotiated partnering that works well toward reaching the persons’ goals. Professionals being in a position of leadership in negotiated partnering have to consider both users and family members as resources and need to be open and flexible for discussions and negotiations with understandings about the perspectives of users and family members in order to make family support as positive as possible [21]. Negotiations among all participants in the situations of clinical services regarding different understandings about the meanings of MHSA problems and situations as well as about different contributions required of various participants were critical in progressing through the clinical process [19]. In negotiated partnering, it is critical that all participants (i.e., users and professionals in this context) are seen as equals, but at the same time that the professionals need to uphold the users’ preferences in partnerships as the primary orientation. Negotiations will always need to be carried out with the preferences for the perspectives and aims of the user.

### Accommodating user-participation

The user’s participation is the foundation of any collaborative endeavor. User-participation encompasses the person being engaged in every aspect of the clinical, therapeutic process especially participating in all activities within it. User-participation means the person’s active involvement in actions as an engaged participant. However, professionals need to be active in promoting and accommodating user-participation to occur in clinical encounters. As the user-participation and user-knowledge are the legitimate base of actions, one simple way for accommodating user participation in collaborative work is a “guideline” reported by Sundet [34] for securing the position of the service user perspectives and participation. This states “… when a disagreement on how to proceed with the therapeutic work arises between service users and therapists, a process that gives priority to the service users’ perspectives, ideas, and preferences is set up [34, p. 126]. Accommodating user-participation is about giving space, voice, and determination to the person’s perspectives, understandings and preferences of action. Furthermore, it entails giving the possibility for self-directed realization of ordinary life as a citizen so that the person is able to participate in actions for recovery willingly, fully, and without constraints from the professionals.

### Addressing the tension between help and control

Professionals’ support of users has to address the tensions that are created by “helping” that can be latently configured by power and control. There is a fine breaking point between being helped and being controlled. In being helped one also shows one’s dependence on the other, creating the situation of the possibility of control. Attaining the balance between help and control was shown in a study of the program for therapist–user pairs through the application of a “control-free zone” through which the participants were able to let go of controls, work together for a goal, and participate in activities together [26]. With the emphasis on creating a safe and supportive environment, the participants were able to share thoughts and feelings without constraints. As this was done in a mutual manner between therapists and users, this supported the therapists in letting go of control that is usually embedded in the traditional manners of being a therapist. Doing things together as equals added something positive to the conversations. Using professional knowledge in service provision, therefore, has to be contextualized for individual users with the perspective of multiplicity in meanings and approaches [22]. This theme points to the need for recognition of the tension and for finding ways to reconcile the tension in both users and professionals in order to proceed with service provision rather than being stuck in the tugging war for power and control.

## Negotiated-supportive processes

Supportive processes as a type of collaborative strategy are rooted in the professionals’ understanding and appreciation of the user’s needs, goals, and wants in everyday lives as well as of the user’s difficulties in dealing with the healthcare system, the community, and the society. The processes encompass strategies to support users to attain and maintain active and meaningful lives in being “the users” of MHSA care. This includes professionals’ ways of helping and supporting users to manage and navigate through the mazes, complexities, and difficulties encountered within healthcare systems and social settings of everyday lives. Supportive processes are based on negotiation and alliance between the service user and the professional, and are collaborative as they are oriented to “helping” users from the users’ perspectives, not determined by the professionals as the authority of what is needed by users. Support is framed by the users’ goals, needs, and wants in the context of their recovery and of their lives. Supportive processes in the context of collaborative practice require the involvement of the users and the professionals in a concerted effort to bring about personally and socially active and meaningful lives for the users. We synthesized the themes identified in the studies and elaborated on these themes by re-reading the empirical material extracting five meta-themes: (a) helping in context, (b) coordinating, (c) pulling together, (d) advocating, and (e) availing.

### Helping in context

Helping in context refers to clinical engagements between the user and the professional that are circumscribed by both the user’s everyday living and recovery in the community and social environments, and by the context of clinical services. Professionals having the perspective of supporting person-in-context are essential in the recovery-oriented practice [21], and Borg et al. [18] found that recovery-oriented mental health work needed to attend to power, unemployment, substandard living conditions and homelessness which are contextual issues impinging on users’ recovery. For example, having a home as a secure base did come out as a necessary condition for recovery. Helping in context means both the professional participating in the realization of a satisfying contextual condition for recovery and providing support in the context of the person’s everyday life. Borg and Karlsson [27] showed how working together with the user in her/his home both increases the experience of safety for the user and the possibilities for the professional to get to know the user and her/his life situation better. Working in the user’s home makes completely different demands on professionals with a different dynamics of power and control, requiring the service that assures autonomy for the user.

Helping in context is oriented to supporting users as they adopt four approaches in living everyday life critical for recovery. These four approaches are: (a) having a normal life characterized by living around ordinary people and doing ordinary things of daily living, (b) doing the things of living in spite of the challenges posed by MHSA problems, (c) having the material conditions of life for comfortable and favorable living or developing coping strategies to handle difficulties arising in social situations, and (d) being good to oneself by engaging in activities and situation that created good feelings and satisfaction [20].

### Coordinating

This theme of coordinating is oriented to supporting users as they encounter the complexities in the healthcare processes and systems. Users of the MHSA services have to navigate through a network of a complex service system, to interact with various healthcare personnel, and to deal with various choices that address different aspects of their needs. One of the critical issues with which the professionals were concerned was the service users’ needs for help in navigating through the bureaucracy of the health care system [28]. Professionals were often engaged in coordinating for users as users found the dynamics of the healthcare system incomprehensible, and needed the professional providers’ insider-knowledge. Coordinating involved helping users to understand the dynamics of services and to have access to various available resources as the parents of young adult users found the system incomprehensible [30]. Parents found it difficult to understand who the right persons were and how different services were organized for their children’s mental health and substance abuse care. Coordinating involves both the willingness of professionals’ engagement in “managing the care” in addition to “providing clinical services” and the users’ acknowledgments for help in their navigation within the services and maneuvering the services and resources to their benefit.

### Pulling together

Pulling together refers to forging together for enriching users’ social life in relation to social participation, active employment, and securing satisfactory home situations. It was critical for professionals to provide support for users’ efforts to function as citizens [20] and being in the community [38]. Having professionals’ support and guidance in the users’ efforts to continue or restore social participation was critical for their journey in recovery. Having the opportunities to partake in activities together by professionals and users as a form of “working together” [26] helped the users to see the ways for social inclusion and building social identity. Professionals’ facilitations to secure homes for users and for social participation were viewed as crucial for recovering from psychosis [18]. Pulling together for users’ active social life meant for the professionals to function as social agents as well as to guide the users in finding the best routes of being active social participants for themselves.

### Advocating

Advocating is one of the key ways of supporting users in the healthcare system. Both from the perspectives of the professionals and of the users, advocating was viewed as critical for users’ participation in the healthcare system. The users were viewed to need advocates in the professionals as the users and their families found it difficult or unable to navigate through the maze of the healthcare system [28]. Users and their family members found it difficult to be their own advocates in seeking appropriate care and services, which pointed to the need for professionals to be advocates for them [30]. Many families that had not been helped by prior treatments also had experienced violations and disparagement in these prior situations, mainly connected to “not being heard” by the professionals and not taken seriously concerning their preferences in the treatment [22]. An important part of the professionals` advocacy was to participate in rectifying these experiences, giving back dignity to the family by securing that they were listened to and taken seriously. For professionals being advocates means having an understanding and appreciation of users’ and their families’ needs, wants, and goals, and being able to articulate these to others in the healthcare system. Conversations between users and professionals for understanding the meanings and wishes are the key to appropriate, valuable advocacy that focuses on the person-in-need, avoiding the use of control and power by professionals in the guise of advocating for users.

### Availing

Availing refers to the idea of the professionals “being there and being available” to people in terms of the users’ perceptions of professionals’ availability for them when needs arise and of the professionals’ factual availability of being present in situations. Availability to provide needed help to users, sometimes even involving the bending or breaking rules and guidelines of what is expected of professionals, was viewed as important by both professionals and users [17]. Availing also means for the professionals to be there as persons to be connected for the users as individuals [28]. Professionals just being there, that is, finding the time to be there for the person was important in order for the person to have the feeling of safety [17]. Sundet [22] also found that “being present where people are” is viewed to be a critical component of helpful therapy. The theme of availing is based on the anticipated needs of users for help and service.

## Discussion

The results of this meta-synthesis provide a set of key strategies applicable to user–professional collaboration in clinical encounters. These 16 meta-themes specifying strategies promotive of collaboration in three different process-types are oriented to (a) *mutual understanding and sharing,* (b) *negotiation, and* (c) *working together*, which are embedded in all meta-themes but are also primarily oriented in specific meta-themes singularly.

The meta-themes of the interactive-dialogic processes are oriented primarily to *mutual understanding and sharing,* which are the foundational base for collaboration [13]. The six meta-themes of this type (maintaining human relationship, walking alongside, information sharing, seizing the present moment, taking the perspective of the other, and alignment and scaffolding) point to strategies for professionals to achieve *mutual understanding* and *sharing*. These are based on the notions of how we understand humanity and personhood. At the base is the idea that humans are unique and, at the same time, share common features and essences. As Arendt states, on one side we are similar to others, on the other side we are different, in the sense that we are unique and irreplaceable [39]. This notion is also found to characterize therapeutic relationships [40] and helpful relationships [41]. Mutual understanding is also based on the acceptance and understanding of differences and uniqueness as experienced by the other and taking the power of such differences to an advantage. Such acceptance and understanding of differences must then become the ways of seeing and experiencing from the position of the other (as in the strategy of taking the perspective of the other). One concept for this is mentalization [42], popularly formulated as the ability to see oneself from the outside and the other from the inside. It is about seeing the other as an intentional, emotional and rational being with one’s unique point of view, emotional states and experiences. Dialogism is all about seeing, retaining and using the difference according to Bakhtin [43, 44]. Flexibility is required for this to occur. Flexibility is supported by and give content to a pluralistic orientation [45, 46]. In addition, the idea of *sharing* involves the openness to exchanging information one holds with the other in order to be on the same foothold. *Sharing* requires an acceptance of the value of the alliance and the freedom from dominance by power and control. Valuing the lived experience of the person is one of the key ingredients for *sharing* [38]. The theme of seizing the moment refers to the adaptive, spontaneous way of dealing with unpredictability in situations by the user and the professional in a concerted approach [47]. On the other hand, the theme of alignment and scaffolding refers to progressive ways of adjusting and aligning professionals with service users [48, 49] and of professionals’ supports to move users toward greater independence [40, 50–53]. The ideas of mutual understanding and sharing are embedded in Habermas’ theory of communicative action in which reaching agreement for coordinated actions among two or more participants is possible only through rationally motivated efforts for mutual approval of the substance of dialogues [54, 55]. This view makes the interactive, dialogic processes as the keystone for collaboration.

The orientations in *negotiation* and *working together* are in the meta-themes that point to two different ways professionals collaborate with service users, which include professionals *working together* with users as partners and as supporters with alliances established through negotiations. *Negotiation* and resulting alliance between the service user and the professional are the features embedded together with the orientation of *working together* in these two process-types*. Negotiation* is inherent as a pre-condition for “*working together”* in collaborative relationships because only through negotiations between participants to identify differences and form alliances that it is possible to move forward in working together. Professionals apply the strategies of the negotiated participatory engagement processes for the partnership role of service users (feedback-informing process, putting differences to work, negotiated partnering, accommodating user participation, and addressing the tension between help and control) to participate in decision-making and planning, therapeutic work, and other clinical activities. On the other hand, professionals are supporters of the person’s efforts in the work of recovery, well-being, and living well with the strategies identified as the negotiated-supportive processes (helping in context, coordinating, pulling together, advocating, and availing). *Working together* means collaborating in the work of recovery for users in concerted, team efforts taking into consideration of persons’ needs and goals, users’ uniqueness, and situational and contextual factors and needs. The feedback-informing process has the formal procedures for shared-decision making and helpful help to users that reflect persons’ needs, goals, and preferences through the application of conversational tools [56]. The strategies in the meta-themes of putting differences to work and negotiated partnering involve “dancing together” in order to move forward for achieving goals. It also refers to the work of de Shazer [57] who developed the concept of “do something different” task adopting Bateson’s [35] definition of information as “different” that makes a difference. It simply involves doing something different rather than what one usually would do. Such different actions could be “different” in the sense of unusual, weird, or strange rather than just being alternatives to the usual. This involves the acceptance of “differentness” and the flexibility to accept and work with differences. The strategy of accommodating user participation directly make it favorable and possible for the person to participate in the work of getting well by applying such techniques as encouragement and positive feedback and also by making the person’s knowledge and needs as the primary grounding for action [38]. Addressing the tension between help and control is critical in clinical encounters as power domination by professionals especially through micro-aggression was seen detrimental to attaining shared decision-making [38]. In order for the person to participate responsibly, it is necessary to address the tension between help and control by balancing the need of users for self-determination and self-efficacy and the application of professional knowledge. The five meta-themes extracted as the supportive process type (helping in context, coordinating, pulling together, advocating, and availing) are strategies for the supportive role of professionals to enhance collaboration. These strategies are based on professionals’ understanding of users’ needs, goals, and preferences, and promote users’ engagement in working toward getting well and recovery less difficult and more positive.

The results of this meta-synthesis have pointed to three distinct process-types of strategies professionals can apply in user–professional collaboration. These processes having the central process of turn-taking as their core point to the interactive nature of the collaborative practice. Collaborative practice in terms of service users/professional collaboration requires the professionals to engage in these processes to be applied as outcome-oriented strategies for service users’ recovery, well-being and living well.

Although this meta-synthesis of the studies by one research group (the CMHSA-USN) has strengths in terms of its findings being coherent and integral framed under the perspective of the research team, it also presents limitations because of the study’s orientation to a specific perspective. The findings are limited to the way the data were analyzed in the original studies and this meta-synthesis under the interpretive perspective. It is possible that a more comprehensive, diversified understanding could have been gained by a meta-synthesis of studies with various perspectives and analytic methods. However, the richness of the findings in the study adds to our knowledge of collaborative strategies, which may not be universally applicable but provide in-depth understandings gained from the analysis taking into accounts the perspectives of research participants. A related limitation is in the generalizability of the findings to other settings that are divergent from the context of the studies in this study, especially in terms of the types of MHSA service settings and of different societal and cultural protocols by which MHSA services are provided and user–professional relationships are shaped in different societies. Norway as the setting of this study is in general mono-cultural and mono-linguistic, and has a national (public) healthcare system. This means that the dynamics of user–professional relationships are shaped by this social context to some extent, and the findings of the study may not have universal generalizability, especially in settings with multicultural characteristics that influence various social and dialogical dynamics in user–professional relationships.

## Concluding remarks

**Fig. 1:**
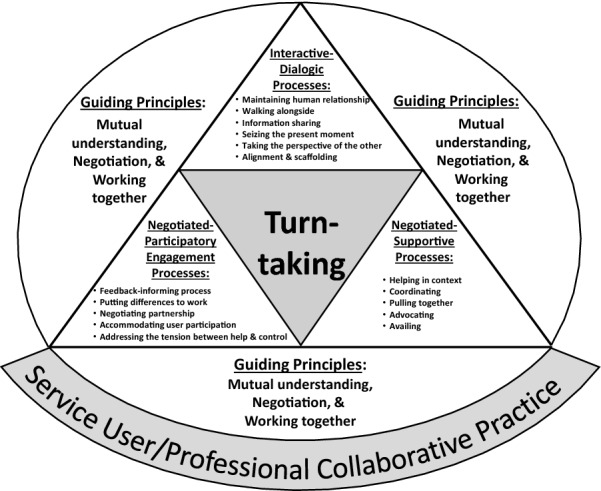
Configuration of the processes in service user/professional collaborative practice

This meta-synthesis of collaborative processes found in community mental health practice shows that collaborative practice involves applying the processes of interactive-dialogic type, negotiated-participatory engagement type, and negotiated-supportive type. These findings point to the possibility of developing a set of repertoires of practice for service users/professional collaboration, especially in community MHSA practice. Figure 1 shows a schematic representation of the findings for service user/professional collaborative practice with the focus on processes.

Turn-taking as the base for all strategies extracted in this study make the interactive nature of user–professional collaboration as the essential feature. The core of the clinical process in community mental health practice, both in terms of singular clinical encounters and continuing clinical relationships, is oriented principally to the goal of addressing the user’s MHSA problems successfully with recovery-orientation. User–professional collaboration is a critical aspect of this clinical process. Although the user–professional collaboration requires the full participation of both the person and the professional in this relational process, it is the responsibility of professionals to establish a safe context and shape this relationship to “be collaborative” by applying appropriate and skillful interactive strategies. The 16 strategies identified as the meta-themes of three process types with the orientations in mutual understanding, negotiation, and working together in this analysis can constitute the contents of the toolbox for collaborative practice in MHSA care.

## Data Availability

The data for this paper are those associated with the published articles identified in the paper. No new data was generated for this study. The datasets analyzed in this study are available from the corresponding authors of the original papers listed in Table 1 upon reasonable request.

## Abbreviations

MHSA: Mental health and substance abuse
The CMHSA-USN: The Centre for Mental Health and Substance Abuse at University of Southeastern Norway
OMFS: The Outcome Monitoring Feedback Systems

## References

[1] Health Canada. Interprofessional education for collaborative patient-centred practice. Health Canada. Health human resources strategy—Interprofessional education. 2004. http://www.hcsc.gc.ca/hcs-sss/hhr-rhs/straeg/interprof/accomp-9_e.html. Assessed 20 Feb 2019.

[2] World Health Organization (WHO). Framework for action on interprofessional education & collaborative practice. Geneva: WHO; 2010. http://111.who.int/hrh/resources/framework_action/en. Assessed 15 Aug 2019.

[3] Meld. St. 47. Samhandlingsreformen Rett behandling – på rett sted – til rett tid. Oslo: Helse- og omsorgsdepartementet. 2008–2009.

[4] UN Human Rights Council. Report of the Special Rapporteur on the right of everyone to the enjoyment of the highest attainable standard of physical and mental health, A/HRC/35/21. 2017. https://www.refworld.org/docid/593947e14.html. Accessed 15 Aug 2019.

[5] Abma TA, Broerse JE (2010). Patient participation as dialogue: setting research agendas. Health Expect.

[6] Seikkula J, Arnkil TE (2006). Dialogical meetings in social networks.

[7] Clarke HF, Mass H (1998). Comox Valley Nursing Centre: from collaboration to empowerment. Public Health Nurs.

[8] Karlsson B, Borg M (2017). Recovery. Tradisjoner, fornyelser og praksiser.

[9] Davidson L (2003). Living outside mental illness. Qualitative studies of recovery in schizophrenia.

[10] Slade M (2009). Personal recovery and mental health illness. A personal guide for mental health professionals.

[11] Baggs JG, Schmitt MH (1997). Nurses’ and resident physicians’ perceptions of the process of collaboration in an MICU. Res Nurs Health.

[12] de Stampa M, Vedel I, Bergman H, Novella JL, Lechowski L, Ankri J (2013). Opening the black box of clinical collaboration in integrated care models for frail, elderly patients. Gerontologist.

[13] Ness O, Karlsson B, Borg M, Biong S, Sundet R, McCormack B (2014). Towards a model for collaborative practice in community mental health care. Scand Psychol.

[14] Sandelowski M, Docherty S, Emden C (1997). Focus on qualitative methods. Qualitative metasynthesis: issues and techniques. Res Nurs Health.

[15] Noblit GW, Hare RD (1988). Meta-ethnography: synthesizing qualitative studies.

[16] Noblit GW. How qualitative (or interpretive or critical) is qualitative synthesis and what we can do about this? A public lecture given at the University of Edinburgh by Professor W. Noblit, University of North Carolina at Chapel Hill; 2016. http://emergenceproject.org/wp-content/uploads/2016/09/How-qualitative.pdf. Accessed 10 Sept 2019.

[17] Borg M, Kristiansen K (2004). Recovery-oriented professionals: helping relationships in mental health services. J Ment Health.

[18] Borg M, Sells D, Topor A, Mezzina R, Marin I, Davidson L (2005). What makes a house a home. The role of material resources in recovery from severe mental illness. Am J Psychiatr Rehabil.

[19] Karlsson B, Borg M, Sjølie H (2008). Om krisehåndtering og hjemmebehandling i psykisk helsevern. Forskning.

[20] Borg M, Davidson L (2008). The nature of recovery as lived experience. J Ment Health.

[21] Borg M, Karlsson B, Lofthus AM, Davidson L (2011). “Hitting the wall”: lived experiences of mental health crises. Int J Qual Stud Health Well-being.

[22] Sundet R (2011). Collaboration: family and therapists’ perspectives of helpful therapy. J Marital Fam Ther.

[23] Ervik R, Sælør KT, Biong S (2012). Å gripe øyeblikket. Tidsskrift for psykisk helsearbeid.

[24] Borg M, Haugård E, Karlsson B (2012). “Uten oss går det ikke” –pårørendes erfaringer med psykisk krise. Nordisk Tidsskrift for Helseforskning.

[25] Sundet R (2012). Therapist perspectives on the use of feedback on process and outcome: patient-focused research in practice. Can Psychol.

[26] Karlsson B, Borg M, Revheim T, Jonassen R (2013). “To see each other more like human beings…from both sides”. Patients and therapists going to a study course together. Int Pract Dev J.

[27] Borg M, Karlsson B (2013). Hjemmet som samarbeidsarena – muligheter og begrensninger i lokalbasert psykisk helsearbeid. Tidsskrift for psykisk helsearbeid.

[28] Ness O, Borg M, Semb R, Karlsson B (2014). “Walking alongside:” collaborative practices in mental health and substance use care. Int J Ment Health Syst.

[29] Sundet R (2014). Patient-focused research supported practices in an intensive family therapy unit. J Fam Ther.

[30] Ness O, Borg M, Semb R, Topor A (2016). “Negotiating partnerships:” parents ‘experiences of collaboration in community mental health and substance abuse services. Adv Dual Diagn.

[31] Sundet R, Kim HS, Ness O, Borg M, Karlsson B, Biong S (2016). Collaboration: suggested understandings. Aust NZ J Fam Ther..

[32] Soggiu AS, Biong S (2017). Samarbeid noen dør med: Om sosialarbeideres erfaringer med klienter som dør i overdose. Tidsskrift for psykisk helsearbeid.

[33] Ness O, Kvello Ø, Borg M, Semb R, Davidson L (2017). “Sorting things out together”: young adults’ experiences of collaborative practices in mental health and substance use care. Am J Psychiatr Rehabil.

[34] Sundet R, Tilden T, Wampold BE (2017). Feedback as means to enhance client-therapist interaction in therapy. Routine outcome monitoring in couple and family therapy. The empirically informed therapist.

[35] Bateson G, Donaldson RE (1991). Mind/environment. A sacred unity. Further steps to an ecology of mind.

[36] Stern DN (1998). The interpersonal world of the infant. A view from psychoanalysis and developmental psychology.

[37] Together Sennett R (2013). The rituals, pleasures & politics of collaboration.

[38] Borg M, Karlsson B, Kim HS (2009). User involvement in community mental health services—principles and practices. J Psychiatr Ment Health Nurs.

[39] Arendt H (1998). The human condition.

[40] Norcross JC, Lambert MJ (2018). Psychotherapy relationships that work III. Psychotherapy.

[41] Ljungberg A, Denhov A, Topor A (2015). The art of helpful relationships with professionals: a meta-ethnography of the perspective of persons with severe mental illness. Psychiatr Q.

[42] Fonagy P, Bateman AW (2006). Mechanisms of change in mentalization-based treatment of BPD. J Clin Psychol.

[43] Bakhtin MM (Emerson C, Holquist M, translators). The dialogic imagination: four essays. London: University of Texas Press; 1981.

[44] Bakhtin MM (Emerson C, translator, editor). Problems of Dostoevsky’s poetics. Minneapolis: University of Minnesota Press; 1984. 10.5749/j.ctt22727z1.

[45] Cooper M, McLeod J (2011). Pluralistic counselling and psychotherapy.

[46] McLeod J, Sundet R, Lindstad TG, Stänicke E, Valsiner J (2020). Working with stuckness in psychotherapy; bringing together the bricoleur-model and pluralistic practices. Respect for thought; Jan Smedslund’s legacy for psychology.

[47] Borg M, Karlsson B, Kim HS (2010). Double helix of research and practice—developing a practice model for crisis resolution and home treatment through participatory action research. Int J Qual Stud Health Well-being.

[48] Baldwin SA, Wampold BE, Imel ZE (2007). Untangling the alliance-outcome correlation: exploring the relative importance of therapist and patient variability in the alliance. J Consult Clin Psychol.

[49] McLeod J (2018). Pluralistic therapy. Distinctive features.

[50] Vygotsky L (1978). Mind in society: the development of higher psychological processes.

[51] Vygotsky L (1986). Thought and language.

[52] White M (2011). Narrative practice. Continuing the conversations.

[53] Lambert MJ, Shimokawa K (2011). Collecting client feedback. Psychotherapy.

[54] Harbermas J (McCarthy TA, translator). Theory of communicative action. Volume One: Reason and rationalization of society. Boston: Beacon Press; 1984. **(Originally published in 1981)**.

[55] Harbermas J (McCarthy TA, translator). Theory of communicative action. Volume Two: Lifeworld and system: a critique of functionalist reason. Boston (MA): Beacon Press; 1987. **(Originally published in 1981)**.

[56] Olkowska A, Sundet R, Karlsson B (2018). Kan klient- og resultatstyrt praksis (KOR) bidra til økt brukermedvirkning og med dette til bedrings- og mestringsprosesser i terapi?. Fokus på familien.

[57] De Shazer S (1985). Keys to solutions in brief therapy.

